# HLA-DPB1*05:01 and HLA-A*11:01 Is Associated with Adverse Drug Reactions to Isoniazid and Rifampin for Treatment of Latent Tuberculosis Infection in South Korea

**DOI:** 10.3390/jcm13123563

**Published:** 2024-06-18

**Authors:** Bomi Kim, Jungok Kim, Sun-Young Yoon, Hae Suk Cheong, Min-Jung Kwon, Joon-Sup Yeom, Han-Na Kim, Eun-Jeong Joo

**Affiliations:** 1 Division of Infectious Diseases, Department of Medicine, Kangbuk Samsung Hospital, Sungkyunkwan University School of Medicine, Seoul 03181, Republic of Korea; bomi.id.kim@samsung.com (B.K.); hs.cheong@samsung.com (H.S.C.); 2 Division of Infectious Diseases, Department of Internal Medicine, Chungnam National University School of Medicine, Daejeon 35015, Republic of Korea; jungok37@gmail.com; 3 Divisions of Allergy and Pulmonology, Department of Internal Medicine, Chungnam National University School of Medicine, Daejeon 35015, Republic of Korea; ggulcha2000@naver.com; 4 Department of Laboratory Medicine, Kangbuk Samsung Hospital, Sungkyunkwan University School of Medicine, Seoul 03181, Republic of Korea; mjk.kwon@samsung.com; 5 Department of Internal Medicine, Yonsei University College of Medicine, Seoul 03722, Republic of Korea; joonsup.yeom@yuhs.ac; 6 Department of Clinical Research Design & Evaluation, Samsung Advanced Institute for Health Sciences & Technology, Sungkyunkwan University, Seoul 06355, Republic of Korea; 7 Biomedical Statistics Center, Research Institute for Future Medicine, Samsung Medical Center, Seoul 06351, Republic of Korea

**Keywords:** adverse drug reactions, drug hypersensitivity, HLA-A*11:01 antigen, HLA-DPB1*05:01 antigen, latent tuberculosis

## Abstract

**Background:** Screening and treating healthcare workers (HCWs) for latent tuberculosis infection (LTBI) are essential for tuberculosis (TB) infection control. Adverse drug reactions (ADRs) to anti-TB drugs present challenges to patient safety and treatment completion. **Objective:** This study investigated the association between human leukocyte antigen (HLA) alleles and the risk of ADRs, especially drug hypersensitivity (DHS) and hepatotoxicity, in HCWs with LTBI receiving isoniazid (INH) and rifampin (RIF) therapy. **Methods:** Korean HCWs with LTBI who received a 3 month INH and RIF regimen were included in this study. *HLA* genotyping was performed on HCWs who experienced ADRs during treatment, as well as the control group consisted of individuals who did not develop ADRs. **Results:** Of the 67 patients, 29 (43.2%) experienced ADRs during INH and RIF therapy. The HLA-A*11:01 allele was more frequent in patients with DHS without hepatotoxicity (DSH+/H−) compared to the control group (DHS−/H−) (4/9, 44.4% vs. 3/38, 7.9%; odd ratio [OR], 8.554; 95% confidence interval [CI], 1.415–59.869; *p* = 0.018). Conversely, HLA-DPB1*05:01 was associated with an increased risk of hepatotoxicity regardless of DHS (10/20, 50% vs. 5/38, 13.2%; OR, 5.323; 95% CI, 1.493–21.518; *p* = 0.011). In the DHS with hepatotoxicity group (DHS+/H+), HLA-DPB1*05:01 was present in a higher proportion (3/5, 60% vs. 5/38, 13.2%; OR, 8.912; 95% CI, 1.110–92.993; *p* = 0.037), whereas HLA-A*11:01 was not observed in this group. **Conclusions:** The HLA-A*11:01 allele was associated with an increased risk of DHS without hepatotoxicity, whereas the HLA-DPB1*05:01 allele was associated with an increased risk of hepatotoxicity.

## 1. Introduction

Latent tuberculosis infection (LTBI) refers to an immune response resulting from a previous *Mycobacterium tuberculosis* infection without any clinical signs of active tuberculosis (TB). Although individuals with LTBI are not contagious, 5–15% eventually develop active TB [1]. Healthcare workers (HCWs) are at a high risk of acquiring *M. tuberculosis* infections due to occupational exposure to patients infected with TB. Moreover, HCWs with LTBI are at risk of transmitting TB to other HCWs and patients. To combat TB, South Korea has implemented various measures, including the Korean National TB Elimination Project and the development of guidelines for diagnosing and managing LTBI in healthcare settings [2,3].

HLA (Human Leukocyte Antigen) is a key component of the immune system, involved in regulating the body’s immune response. Variants in *HLA* genes can affect the binding specificity of HLA proteins, influencing how the immune system recognizes and responds to different antigens. This genetic variability can lead to differences in individual susceptibility to adverse drug reactions (ADRs) [4].

ADRs are unintended and harmful reactions to medications, which can range from mild symptoms like gastrointestinal intolerance to severe conditions such as hepatotoxicity or drug hypersensitivity (DHS) [5]. Research has shown that certain *HLA* variants are associated with an increased risk of ADRs [4]. Understanding this relationship between *HLA* and ADRs is critical for determining why some patients experience severe reactions while others do not, highlighting the importance of considering genetic factors in drug safety and personalized medicine.

The standard treatment regimen for latent tuberculosis infection (LTBI) includes isoniazid (INH) for 9 months, rifampin (RIF) for 4 months, or a combination of INH and RIF for 3 months [6]. However, these treatments can lead to ADRs, which may necessitate the interruption or discontinuation of LTBI therapy. Given the significance of genetic associations in influencing individual susceptibility to ADRs, our study focuses on investigating the relationship between *HLA* alleles and the risk of ADRs in patients treated with INH and RIF. Our primary objective was to identify genetic variants that could potentially predict the occurrence of ADRs, with a particular emphasis on hepatotoxicity and DHS, which are the most common ADRs associated with these medications.

## 2. Materials and Methods

### 2.1. Enrollment of Study Population

HCWs at Kangbuk Samsung Hospital are required to undergo LTBI screening before being assigned to their respective working units, as mandated by the Tuberculosis Prevention Law enacted in August 2016. Since February 2017, all hospital employees underwent an interferon-gamma release assay (IGRA) for LTBI screening. In cases where a HCW is diagnosed with LTBI, a treatment regimen of daily INH plus RIF for 3 months is advised [5]. It should be noted that rifapentine is not currently available in the Korean pharmaceutical market yet [6]. According to the LTBI management protocol, the patients regularly visited the Infectious Diseases Clinic at baseline and at 2 weeks, 6 weeks, and 3 months until the end of treatment. During each visit, the participants were interviewed regarding any symptoms and underwent ADR examinations conducted by infectious disease specialists. Blood tests, including complete blood counts and serum biochemical parameters, were performed at each visit to monitor the occurrence of ADRs.

Eligible participants were recruited based on the following criteria: (a) normal baseline serum aspartate transaminase (AST), alanine transferase (ALT), total bilirubin, and creatinine levels; (b) follow-up blood sampling for complete blood count and serum biochemical parameters; (c) at least two visits for the evaluation of anti-TB medication side effects, (d) no concurrent medication use; and (e) consent to participate in this study. Serological tests for hepatitis B and C viruses were performed to exclude chronic viral hepatitis.

This study was approved by the Institutional Review Board of the Kangbuk Samsung Hospital (KBSMC 2017-07-041-009, registered on 9 August 2017). The study reviewed records of HCWs at Kangbuk Samsung Hospital diagnosed with LTBI via IGRA testing, who underwent treatment with INH and RIF from 1 February 2017 to 1 December 2017. This review focused on ADRs to the treatment and related blood test outcomes. Recruitment of participants for *HLA* genotyping occurred between 16 October and 1 December 2017, with written consent secured and duly recorded. Since the study did not involve minors, the need for parental or guardian consent was not relevant.

### 2.2. Definition of ADRs

ADRs were defined as an appreciably harmful or unpleasant reaction, resulting from an intervention related to the use of a medicinal product, which predicts hazard from future administration and warrants prevention or specific treatment, or alteration of the dosage regimen, or withdrawal of the product [7].

DHS is unpredictable and restricted to vulnerable subpopulations. To classify the severity of DHS, a grading system consisting of three grades correlating with mild, moderate, and severe reactions based on the clinical features of anaphylaxis was used [8]. Mild-grade reactions were limited to skin manifestations, including cutaneous and subcutaneous conditions such as erythema, urticaria, periorbital edema, and angioedema. Moderate-grade reactions involve symptoms in at least two organs or systems such as the cardiovascular, respiratory, and gastrointestinal systems. Severe-grade reactions were characterized by clinical features such as hypoxia, hypotension, or neurologic compromise, often presenting with cyanosis or saturation of peripheral oxygen (SpO2) of ≤92%.

Drug-induced liver injury (DILI) or hepatitis was suspected when the ALT level was ≥5 times the upper limit of normal in the absence of symptoms, or ≥3 times the upper limit of normal in the presence of hepatitis symptoms [5]. The severity of toxicity was classified as mild if the ALT level was <5 times the upper limit of normal, moderate if it was 5–10 times the normal limit, and severe if it was >10 times the normal limit. In this study, the term ‘hepatotoxicity’ is used to describe DILI.

### 2.3. Diagnosis of LTBI

The QuantiFERON-TB Gold in tube (QFT-GIT; Qiagen, Hilden, Germany) is an in vitro diagnostic test which measures the IFN-gamma released by cell-mediated immune responses to peptide antigens in heparinized whole blood. The antigens used are a peptide cocktail simulating ESAT-6, CFP-10, and TB7.7 proteins. The QFT-GIT assay uses specialized blood collection tubes with a Nil tube, a TB Antigen tube, and a Mitogen tube, and blood collection was performed in that order. Incubation of collection of the blood occurs in the tubes for 16 to 24 h at 37 °C, after which the tubes are centrifuged, the plasma is harvested, and the amount of IFN-γ is measured using ELISA (Dynex DS2 system, Chantilly, VA, USA). A QFT-GIT assay is considered to be positive for an IFN-γ response to the TB Antigen tube that is significantly above the Nil IFN-γ IU/mL value. A low response to Mitogen (<0.5 IU/mL) indicates an indeterminate result when a blood sample also has a negative response to the TB antigens. The results of the QFT-GIT assay in our study were interpreted, as described in the published guidelines [9]. The Laboratory Medicine Department at Kangbuk Samsung Hospital is accredited and participates annually in inspections and surveys by the Korean Society of Laboratory Medicine and the Korean Association of Quality Assurance for Clinical Laboratories.

### 2.4. HLA Genotyping

Peripheral blood samples were collected from all participants and stored in ethylenediaminetetraacetic acid (EDTA) tubes. The samples were sent to Macrogen, Inc. (Seoul, Republic of Korea) for whole-exome sequencing (WES) and genomic DNA extraction. The library was prepared using the SureSelect V6-Post kit (Agilent, Santa Clara, CA, USA) and was sequenced on NovaSeq6000 (Illumina, San Diego, CA, USA) at 100× read depth. Allele-level genotypes for six *HLA* loci (*HLA* class I [A, B, and C] and class II [*DPB1*, *DQB1*, and *DRB1*]) were obtained from WES data using the HLAscan pipeline [10]. For *HLA* typing using WES data, HLAscan, an alignment-based multistep *HLA* typing method that uses short sequence reads of next-generation sequencing (NGS) data, was used. The method was approved for higher accuracy of *HLA* typing compared to the results obtained using previously reported software for *HLA* typing (PyHLA, v1.1) [11]. A map of the *HLA* region was obtained from the International ImMunoGeneTics Project/Human Leukocyte Antigen (IMGT/HLA) database (https://www.ebi.ac.uk/ipd/imgt/hla/ accessed on 1 July 2018).

### 2.5. Statistical Analysis

Demographic and clinical variables were presented as medians with interquartile ranges (IQR) for continuous variables and as N (%) for categorical variables. The Kruskal–Wallis test was used to analyze the continuous variables, and chi-squared test or Fisher’s exact test was used for the categorical variables. A *p*-value of less than 0.05 was considered to be statistically significant. Statistical analysis was performed using SPSS version 28.0 (SPSS Corp., Armonk, NY, USA). The frequencies of *HLA* class I and class II genotypes in the patient group were investigated, and the frequency of each allele in patients with any ADRs among DHS and hepatotoxicity (H) was compared with controls without ADRs (DHS−/H−). We performed a basic allele-based Fisher exact test for the 6 *HLA* loci, comparing allele frequency difference between case and control groups. We assumed a dominant effect of the allele at the *HLA* locus, which was the genotypic test with 1 degree of freedom (df), and compared them as the prevalence according to the ADRs to INH and RIF. Statistical analyses of the *HLA* associations were performed using a two-tailed Fisher’s exact test. The significance threshold was set with a false positive rate (FDR)-adjusted *p* < 0.05, following the Benjamini–Hochberg correction. Sensitivity, specificity, positive predictive value (PPV), and negative predictive value (NPV) were calculated using the moonBook package from R (Version 4.0.2).

## 3. Results

### 3.1. Study Participants

**Figure 1:**
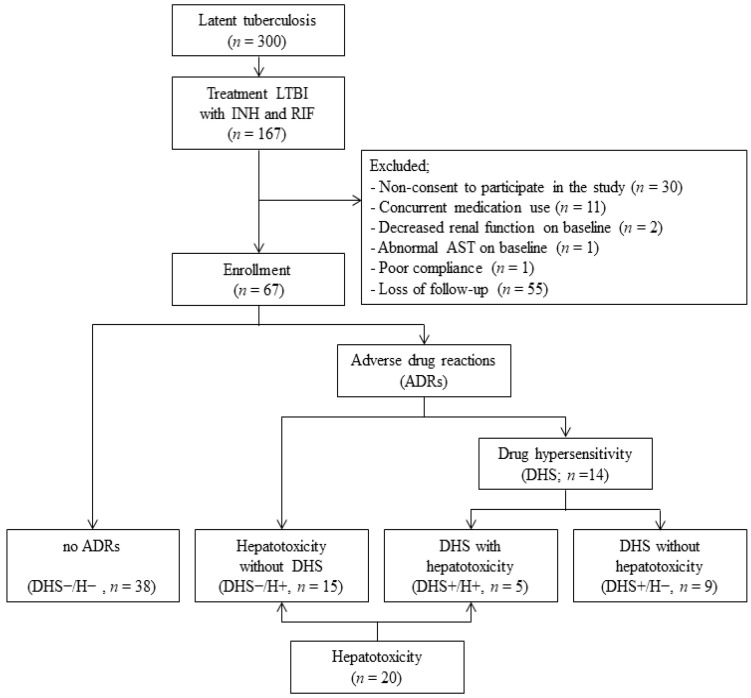
The enrollment of study participants with LTBI.

Between January and July 2017, 300 HCWs were diagnosed with LTBI. Among the patients with LTBI, 55% (*n* = 167) received daily INH and RIF. Figure 1 provides an overview of the enrollment process of the study population. Of the 167 patients receiving anti-LTBI medications, 100 were excluded, and among them, 55 were lost to follow-up. Ultimately, 67 participants were included in this study. To evaluate the association between ADRs and *HLA* alleles, the enrolled patients were categorized into four subgroups: no ADRs with complete LTBI treatment (DHS−/H−, *n* = 38), DHS without hepatotoxicity (DHS+/H−, *n* = 9), DHS with hepatotoxicity (DHS+/H+, *n* = 5), and hepatotoxicity without DHS (DHS−/H+, *n* = 15).

### 3.2. Baseline Characteristics

Of the 67 patients, 29 (43.2%) experienced ADRs during LTBI treatment (Table 1). Among them, 14 (20.8%) had DHS and 20 (29.8%) had hepatotoxicity (H). Five patients exhibited both clinical manifestations. All patients were Korean and ranged between 24 and 60 years of age. Most participants were female. The DHS−/H+ group tolerated and completed the LTBI treatment. Compared with the DHS−/H+ group, the DHS+/H+ group had higher peak levels of elevated hepatic enzymes. All patients in both groups experienced mild hepatotoxicity, and none developed fulminant liver failure or injury. Most cases of DHS (85.7%) occurred within 30 days of initiating medication, and no severe DHS or anaphylactic reactions were observed during the study period. None of the patients had previous exposure to INH or RIF. Re-challenge was attempted in only three patients, all of whom developed skin manifestations. Among the 14 patients with DHS, 11 (16.4%, 11/67) discontinued LTBI treatment. Table 2 provides descriptions of the clinical characteristics of DHS.

### 3.3. Association of the HLA Allele and the Risk of ADRs to RIF and INH

The frequencies of *HLA* alleles carried by >5% of the subjects among the 6 *HLA* loci (*HLA* class I [A, B, and C], and class II [DPB1, DQB1, DRB1]) were compared among the subgroups (Supplementary Tables S1–S3). At the *HLA-A* locus, HLA-A*11:01 was found to be the most frequent allele in the DHS+/H− group, with an allele frequency of 27.8%. The most common *HLA-DPB1* allele was DPB1*05:01, with an allele frequency of 33.3%, 60%, and 46.7% in the DHS+/H−, DSH+/H+, and DHS−/H+ groups, respectively. In the allele-based association test, we found that the two *HLA* alleles, HLA-A*11:01 and HLA-DBP1*05:01, were significantly associated with ADRs to INH and RIF.

Next, we compared the two *HLA* alleles’ prevalence according to the subtype of ADRs to INH and RIF. The HLA-A*11:01 prevalence was higher in subjects with DHS+/H− than in the control group (DHS−/H−) (4/9, 44.4% vs. 3/38, 7.9%; odds ratio [OR], 8.554; 95% confidence interval [CI], 1.415–59.869; *p* = 0.018) (Table 3). As a genetic marker for predicting DHS+/H−, HLA-A*11:01 exhibited a sensitivity of 44.4% and specificity of 92.2%. The positive predictive value of HLA-A*11:00 for DHS+/H− was 57.1%, and the negative predictive value was 87.5%.

Regarding predicting hepatotoxicity irrespective of DHS, the HLA-DPB1*05:01 allele was present in a higher proportion in the hepatotoxicity group compared to the DHS−/H− group (10/20, 50% vs. 5/38, 13.2%; OR, 5.323; 95% CI, 1.493–21.518; *p* = 0.011). As a genetic marker for predicting hepatotoxicity irrespective of DHS, HLA-DPB1*05:01 exhibited a sensitivity of 50% and a specificity of 86.8%. The positive predictive value of HLA-DPB1*11:00 for hepatotoxicity was 66.7%, and the negative predictive value was 76.7%. Among subgroups within hepatotoxicity, HLA-DPB1*05:01 was significantly associated with the DHS+/H+ group (3/5, 60% vs. 5/38, 13.2%; OR 8.912; 95% CI, 1.110–92.993; *p* = 0.037), whereas HLA-A*11:01 was not observed in this group. In this group, HLA-DPB1*05:01 exhibited a sensitivity of 60% and a specificity of 86.8%. The positive predictive value of HLA-DPB1*11:00 was 37.5%, and the negative predictive value was 94.3%, indicating that these genetic biomarkers are necessary but not sufficient for the triggering of such immunoallergic events.

## 4. Discussion

In the present study, the association between *HLA* alleles and the risk of ADRs, particularly DHS and hepatotoxicity, in patients receiving INH and RIF therapy for LTBI, was investigated. Our findings revealed a significant association between specific *HLA* alleles, HLA-A*11:01 and HLA-DPB1*05:01, and the occurrence of ADRs.

We observed a higher frequency of the HLA-A*11:01 allele in the DHS+/H− group and HLA-DPB1*05:01 alleles in the hepatotoxicity group, irrespective of DHS, than in the control group without any ADRs (DSH−/H−). The sensitivity and specificity of HLA-A*11:01 in predicting DHS+/H− were determined to be 44.4% and 92.2%, respectively, whereas the sensitivity and specificity of HLA-DPB1*05:01 in predicting hepatotoxicity were determined to be 50% and 86.8%, respectively. These findings suggest that the presence of HLA-A*11:01 and HLA-DPB1*05:01 is associated with increased susceptibility to ADRs in patients receiving INH and RIF therapy. Therefore, HLA-A*11:01 may serve as a potential genetic marker for predicting DHS without hepatotoxicity, and HLA-DPB1*05:01 may serve as a marker for hepatotoxicity.

Recent research revealed genetic variants in the *HLA* region that are associated with ADRs to various drugs [4]. Abacavir (HLA-B*57:01), carbamazepine (HLA-A*31:01 and HLA-B*15:02), allopurinol (HLA-B*58:01), and lamotrigine (HLA-A*31:01 and HLA-B*15:02) are well-known drugs that induce *HLA*-associated ADRs in cutaneous DHS [12,13,14,15,16,17]. Amoxicillin-clavulanate (DRB1*15:01-DQB1*06:02) and flucloxacilin (HLA-B*57:01) has been associated with DILI [18,19]. However, data on the association among *HLA* genotyping, ADRs, and anti-TB medications are limited. A previous study reported that HLA-C*04:01 was associated with drug-induced hypersensitivity syndrome (also known as drug reactions with eosinophilia and systemic syndromes [DRESS]) in a Korean population receiving standard anti-TB drugs [20]. Another study investigated HLA-DQB1*02:01, in combination with the absence of HLA-DQA1*01:02, as a risk factor for DILI in an Indian population [21]. In comparison to previous studies, our study identified associations between specific *HLA* alleles and the clinical manifestations of ADRs in patients receiving INH and RIF therapy, namely, the HLA-A*11:01 allele in DHS without hepatotoxicity and the HLA-DPB1*05:01 allele in DHS with hepatotoxicity. Notably, none of the participants in the DHS+/H+ group carried the HLA-A*11:01 allele. This observation may be attributed to the small number of patients in our study, and further evaluation in a larger study is required to explore potential genetic factors associated with ADRs.

The precise mechanisms underlying ADRs are not well understood. Allergic drug reactions, classified as type B ADRs, include immune-mediated hypersensitivity reactions that interact with the major histocompatibility complex (MHC), known as the *HLA* gene locus on chromosome 6 in humans [22]. *HLA* genes encode proteins involved in antigen presentation to T cells, which elicit immune responses by binding to self-derived peptides or those from foreign antigens. Variations in highly polymorphic *HLA* genes ultimately influence susceptibility to autoimmune diseases, drug-induced hypersensitivity, and other immune-related responses [4]. In specific genetic populations, the HLA-DPB1*05:01 allele is found at a high frequency in Asia, including South Korea, but is rare in other ethnic populations [23,24,25]. Some studies have suggested an association between *HLA* alleles and autoimmune diseases in Asian populations. Studies on Japanese populations have indicated that DPB1*05:01 is associated with Asian-type multiple sclerosis, rheumatoid arthritis (RA), and systemic lupus erythematosus (SLE) [26,27,28]. It has also been associated with neuromyelitis optica in the Southern Han Chinese population [29]. However, the role of HLA-A*11:01 in ADRs has rarely been investigated in Asian populations [30]. Recent studies have indicated that HLA-A*11:01 is associated with severe sulfonamide-related cutaneous adverse reactions (SCARs) in Japanese patients [31]. Furthermore, it is notable that specific allele frequencies can change over time, owing to factors such as population migration, intermarriage, and natural selection. Therefore, our findings contribute to the growing body of evidence on the association between *HLA* and ADRs, considering ethnic populations with different frequencies of allele presentation.

Among LTBI treatment regimens, the 3-month INH-rifamycin (RIF or rifapentine) regimen is preferred because of its similar efficacy, shorter duration, and favorable tolerability [6]. DILI can occur with all LTBI treatment agents, but often resolves spontaneously over days to weeks without requiring alteration or discontinuation of therapy. The estimated rate of DILI ranges 2.5–25% when at least one anti-TB drug is administered, with a higher incidence observed when INH is combined with RIF than when INH is administered alone [32]. The exact mechanisms and contributing factors of DILI remain poorly understood, involving possible direct hepatic toxicity of the drugs or their metabolites, as well as immunologically mediated responses, such as DHS [33]. Although the occurrence of DHS in response to anti-TB drugs is relatively limited, it affects approximately 3–4% of patients within the first few weeks after therapy initiation [34]. DHS is a barrier to treatment adherence, and can lead to detrimental outcomes. Unfortunately, the availability of additional tests for identifying DHS, such as skin prick, intradermal, patch, and in vitro tests, is limited. Despite these limitations, studies have demonstrated higher treatment completion rates with prescribed INH-rifamycin short-course combinations [35]. In our study, all patients experienced mild hepatotoxicity and tolerated the continued therapy. However, among the 14 patients with DHS, 11 (78.6%) discontinued LTBI therapy. Future availability of screening tests to detect ADRs can empower patients to make informed decisions and reduce the risk of ADRs by selecting a suitable LTBI treatment regimen.

This study has several limitations. First, the sample size was relatively small, which may have limited the generalizability of our findings. Although we initially anticipated a large cohort, the loss of follow-up patients reduced the study population size. The primary reasons included resignation from employment and personal reasons unrelated to the study. Additionally, some participants lacked understanding of LTBI and were unaware of the need for treatment, leading to reluctance to complete the LTBI therapy even after initiating it. Despite the reduced sample size, we believe our findings remain robust and provide valuable insights. Larger studies involving healthy and diverse populations are required to validate our results. Second, our study focused specifically on *HLA* alleles and their association with ADRs to RIF and INH therapies in the Korean population. Third, one of the major limitations of our study is the issue of multiple comparisons. We did not apply any adjustments for multiple comparisons for the six groups, which can increase the risk of Type I error. Future studies should consider employing more stringent statistical methods to account for multiple testing comparisons and validate these findings in larger, independent cohorts to provide more robust evidence. Forth, the study design was retrospective, and data collection relied on medical records, which may have introduced potential biases. However, efforts have been made to standardize the ADRS monitoring protocols. Finally, the study population had no severe-grade ADRs or hepatotoxicity.

In conclusion, our study highlights the potential role of *HLA* alleles in predicting the occurrence of ADRs, particularly DHS and hepatotoxicity, in patients receiving INH and RIF therapy for LTBI. The HLA-A*11:01 allele was associated with an increased risk of DHS without hepatotoxicity, whereas the HLA-DPB1*05:01 allele was associated with an increased risk of hepatotoxicity. These findings emphasize the importance of *HLA* genotyping in guiding the selection of optimal treatment regimens to minimize the risk of ADRs and complete LTBI treatment in this specific patient population. Further research with large sample sizes and a diverse population is warranted to validate our finding and explore the clinical utility of *HLA* typing in personalized medical approaches for LTBI treatment.

## Figures and Tables

**Table 1:** Baseline characteristics of patients with LTBI with RIF and INH.

Characteristics	ADRs			DHS−/H− (*n* = 38)	*p*-Value
	DHS+/H− (*n* = 9)	DHS+/H+ (*n* = 5)	DHS−/H+ (*n* = 15)		
Age (years)	45 (36.5–52.5)	36 (32.0–41.5)	41 (33.0–47.0)	38.2 (28.0–48.0)	0.275
Female	6 (66.7)	5 (100)	7 (46.7)	30 (62.5)	0.488
Occupation					
Doctor	-	-	4 (26.7)	2 (5.3)	0.116
Nurse	5 (55.6)	5 (100)	3 (20.0)	13 (34.2)	0.008
Support services staff	2 (22.2)	-	5 (33.3)	16 (42.1)	0.264
Office clerk	2 (22.2)	-	3 (20.0)	7 (18.4)	0.841
Liver function test (IU/L)					
Baseline					
AST	19.0 (16.5–22.5)	20.5 (15.3–23.5)	18.0 (16.0–23.0)	18.0 (15.0–22.0)	0.923
ALT	19.0 (14.5–23.0)	14.5 (8.00–20.3)	16.0 (13.8–22.0)	13.0 (10.0–23.0)	0.602
Peak levels during treatment					
AST	18.0 (15.5–23.5)	113.0 (62.5–153.5)	36.0 (26.0–46.0)	18.0 (15.0–21.3)	<0.001
ALT	18.0 (14.5–22.5)	92.0 (78.0–98.0)	39.0 (26.0–59.0)	16.0 (13.8–22.8)	<0.001
Grade of DHS					
Mild	3 (33)	-	NA	NA	
Moderate	6 (67)	5 (100)	NA	NA	
Severe	-	-	NA	NA	
Drug exposure (days)					
≤15	3 (33.3)	2 (40)	NA	NA	1.000
16–30	5 (55.6)	2 (40)	NA	NA	1.000
31–60	-	1 (20)	NA	NA	0.357
61–90	1 (11.1)	-	NA	NA	1.000

RIF: rifampicin; INH: isoniazid; LTBI: latent tuberculosis infection; ADRs: adverse drug reactions; DHS: drug hypersensitivity; H: hepatotoxicity; DHS+/H−: DHS without hepatotoxicity; DHS+/H+: DSH with hepatotoxicity; DSH−/H+: hepatotoxicity without DHS; DSH−/H−: no ADRs; ALT: alanine aminotransferase; AST: alanine aminotransferase; NA: not available; SD: standard deviation. The data are expressed as numbers (%) of patients, unless otherwise indicated. Continuous variables are expressed as median and interquartile range (IQR).

**Table 2:** Demographics of patients who experienced DHS with RIF and INH treatment.

Case No.	Sex	Age (Years)	Occupation	Drug Exposure (Days)	Grade of DHS	Hepatotoxicity (Peak ALT Level [IU/L])	Hospitalization	Completed LTBI Treatment	Re-Challenge for RIF
1	F	55	Nurse	21	Mild: MPE	No		No	No
2	F	45	Office clerk	21	Mild: MPE	No		Yes: INH	No
3	M	46	Engineer	64	Mild: MPE	No		Yes: RIF and INH	Yes: tolerable †
4	F	52	Cook	5	Moderate: MPE and dyspnea	No		No	Yes: MPE after RIF re-exposure
5	F	32	Nurse	14	Moderate: fever, MPE, and cervical lymphadenopathy	No		No	No
6	F	45	Nurse	15	Moderate: fever, MPE, nausea, vomiting, and thrombocytopenia	No	Yes	No	No
7	M	53	Nurse	21	Moderate: fever, MPE, myalgia, nausea, arthralgia, and thrombocytopenia	No		No	No
8	M	35	Office clerk	28	Moderate: MPE, angioedema, and wheezing	No		No	No
9	F	38	Nurse	28	Moderate: fever, MPE, and eosinophilia	No		No	No
10	F	46	Nurse	14	Moderate: MPE, headache and nausea	Yes (104)		No	Yes: MPE after RIF re-exposure
11	F	37	Nurse	14	Moderate: fever, MPE, nausea, headache, and dizziness	Yes (87)	Yes	No	No
12	F	28	Nurse	21	Moderate: fever and MPE	Yes (92)		Yes: INH	No
13	F	36	Nurse	26	Moderate: MPE and nausea	Yes (69)		No	Yes: MPE after RIF re-exposure
14	F	36	Nurse	37	Moderate: MPE and nausea	Yes (217)		No	No

† Patients who completed LTBI treatment with supportive care. RIF: rifampin; INH: isoniazid; DHS: drug hypersensitivity; ALT: alanine aminotransferase; LTBI: latent tuberculosis infection; MPE: maculopapular erythema.

**Table 3:** Association of *HLA* alleles and risk of ADRs in patients with LTBI treatment.

*HLA* Allele	DHS+ (*n* = 14)	DHS+/H− (*n* = 9)	DHS+/H+ (*n* = 5)	DHS−/H+ (*n* = 15)	H+ (*n* = 20)	DHS−/H (*n* = 38)	OR (95% CI) ^†^	*p*-Value
HLA-A*11:01	4 (28.6)	-	-	-	-	3 (7.9)	4.448 (0.806–27.764)	0.075
	-	4 (44.4)	-	-	-	3 (7.9)	8.554 (1.415–59.869)	0.018
	-	-	0 (0)	-	-	3 (7.9)	-	-
	-	-	-	2 (13.3)	-	3 (7.9)	1.808 (0.192–13.213)	0.614
	-	-	-	-	2 (10)	3 (7.9)	1.354 (0.147–9.673)	1
HLA-DPB1*05:01	6 (42.9)	-	-	-	-	5 (13.2)	4.730 (1.124–21.302)	0.050
	-	3 (33.3)	-	-	-	5 (13.2)	3.225 (0.511–18.082)	0.167
	-	-	3 (60)	-	-	5 (13.2)	8.912 (1.110–92.993)	0.037
	-	-	-	7 (46.7)	-	5 (13.2)	5.486 (1.372–24.075)	0.024
	-	-	-	-	10 (50)	5 (13.2)	5.323 (1.493–21.518)	0.011

† The DHS−/H− group was used as the reference group. HLA: human leukocyte antigen; ADRs: adverse drug reactions; LTBI: latent tuberculosis infection; DHS: drug hypersensitivity; H: hepatotoxicity; DHS+/H−: DHS without hepatotoxicity; DHS+/H+: DSH with hepatotoxicity; DSH−/H+: hepatotoxicity without DHS; DSH−/H−: no ADRs; OR: odds ratio; CI: confidence interval. Data are expressed as the sample size (frequency, %) of patients. The statistics were estimated using Fisher’s exact test.

## Data Availability

The authors confirm that the data supporting the findings of this study are available within the article.

## Supplementary Materials

The following supporting information can be downloaded at: https://www.mdpi.com/article/10.3390/jcm13123563/s1, Table S1: Association of *HLA* alleles and risk of DHS without hepatotoxicity in patients with LTBI treatment, Table S2: Association of *HLA* alleles and risk of DHS with hepatotoxicity in patients with LTBI treatment, Table S3: Association of *HLA* alleles and risk of hepatotoxicity without DHS in patients with LTBI treatment.
