# Supplementary material for: HLA-DPB1*05:01 and HLA-A*11:01 Is Associated with Adverse Drug Reactions to Isoniazid and Rifampin for Treatment of Latent Tuberculosis Infection in South Korea
Source: J Clin Med. 2024 Jun 18;13(12):3563. doi: 10.3390/jcm13123563 (PMC11204531; doi:10.3390/jcm13123563)
Supplement: Supplementary file 1 [file jcm-13-03563-s001.zip › jcm-2987861-supplementary.pdf]

**Table S1.** Association of HLA alleles and risk of DHS without hepatotoxicity in patients with LTBI treatment.

| HLA allele | DHS without hepatotoxicity (DHS+/H-) (allele count = 18) | No ADRs (DHS-/H-) (allele count = 76) | OR (95% CI)† | *P*-value | $P_{adj}$-value |
|---|---|---|---|---|---|
| *HLA-A*11:01* | 5 (0.2778) | 3 (0.0395) | 9.3590 (1.99-44.0161) | 0.0059 | 0.0295 |
| *HLA-B*15:01* | 5 (0.2778) | 7 (0.0921) | 3.7912 (1.0419-13.7951) | 0.0491 | 0.1472 |
| *HLA-C*01:02* | 1 (0.0556) | 21 (0.2762) | 0.1541 (0.0193-1.2314) | 0.0624 | 0.2498 |
| *HLA-C*04:01* | 5 (0.2778) | 6 (0.0789) | 4.4872 (1.1912-16.9029) | 0.0327 | 0.2498 |
| *HLA-A*02:01* | 1 (0.0556) | 19 (0.2500) | 0.1765 (0.022-1.4162) | 0.1074 | 0.2686 |
| *HLA-B*51:01* | 4 (0.0222) | 7 (0.0921) | 2.8163 (0.7256-10.9313) | 0.2126 | 0.3189 |
| *HLA-DPB1*05:01* | 6 (0.3333) | 10 (0.1316) | 3.3000 (1.0096-10.7866) | 0.0741 | 0.5190 |

Data are expressed as the number (frequency) of alleles in the population sample (2 × individuals). The statistics were estimated using Fisher's exact test.
† No ADRs (DHS-/H-) group was used as the reference group. $P_{adj}$ values were corrected for multiple comparisons using the Benjamini-Hochberg correction (FDR). Abbreviations: HLA, human leukocyte antigen; LTBI, latent tuberculosis infection; DHS, drug hypersensitivity; ADRs, adverse drug reaction; OR, odds ratio; CI, confidence interval

**Table S2.** Association of HLA alleles and risk of DHS with hepatotoxicity in patients with LTBI treatment.

| HLA allele | DHS with Hepatotoxicity (DHS+/H+) (allele count = 10) | No ADRs (DHS-/H-) (allele count = 76] | OR (95% CI) † | *P*-value | $P_{adj}$-value |
|---|---|---|---|---|---|
| *HLA-DPB1**05:01 | 6 (0.6) | 10 (0.1316) | 9.9000 (2.3707–41.3421) | 0.0023 | 0.0090 |
| *HLA-A**24:02 | 5 (0.5) | 15 (0.1974) | 4.0667 (1.0414–15.8802) | 0.0481 | 0.0962 |
| *HLA-DQB1**05:01 | 4 (0.4) | 10 (0.1316) | 4.4000 (1.0536–18.3743) | 0.0530 | 0.3181 |

Data are expressed as the number (frequency) of alleles in the population sample (2× individuals). The statistics were estimated using Fisher's exact test. † No ADRs (DHS-/H-) group was used as the reference group. $P_{adj}$ values were corrected for multiple comparisons using the Benjamini-Hochberg correction (FDR). Abbreviations: HLA, human leukocyte antigen; LTBI, latent tuberculosis infection; DHS, drug hypersensitivity; ADRs, adverse drug reaction; OR, odds ratio; CI, confidence interval.

**Table S3.** Association of HLA alleles and risk of hepatotoxicity without DHS in patients with LTBI treatment.

| HLA allele | Hepatotoxicity without DHS (DHS-/H+) (allele count = 30) | No ADRs (DHS-/H-) (allele count = 76) | OR (95% CI) † | *P*-value | $P_{adj}$-value |
|---|---|---|---|---|---|
| *HLA-DPB1*05:01* | 14 (0.4667) | 10 (0.1316) | 5.7750 (2.1713–15.3593) | 0.0005 | 0.0026 |
| *HLA-A*02:01* | 2 (0.0667) | 19 (0.2500) | 0.2143 (0.0466–0.9853) | 0.0338 | 0.1689 |
| *HLA-A*24:02* | 10 (0.3333) | 15 (0.1974) | 2.0333 (0.7894–5.2377) | 0.2029 | 0.5072 |
| *HLA-DQB1*03:01* | 7 (0.2333) | 10 (0.1316) | 2.0087 (0.6847–5.8925) | 0.2420 | 0.5647 |
| *HLA-DQB1*03:02* | 1 (0.0333) | 10 (0.1316) | 0.2276 (0.0278–1.8614) | 0.1741 | 0.5647 |
| *HLA-DQB1*05:01* | 7 (0.2333) | 10 (0.1316) | 2.0087 (0.6847–5.8925) | 0.2420 | 0.5647 |
| *HLA-B*07:02* | 4 (0.1333) | 3 (0.0395) | 3.7436 (0.7847–17.8591) | 0.0977 | 0.5859 |

Data are expressed as the number (frequency) of alleles in the population sample (2 × individuals). The statistics were estimated using Fisher's exact test. † No ADRs (DHS-/H-) group was used as the reference group. $P_{adj}$ values were corrected for multiple comparisons using the Benjamini-Hochberg correction (FDR). Abbreviations: HLA, human leukocyte antigen; LTBI, latent tuberculosis infection; DHS, drug hypersensitivity; ADRs, adverse drug reaction; OR, odds ratio; CI, confidence interval.
